# The Maintenance of Established Remote Contextual Fear Memory Requires ERK5 MAP Kinase and Ongoing Adult Neurogenesis in the Hippocampus

**DOI:** 10.1371/journal.pone.0050455

**Published:** 2012-11-26

**Authors:** Yung-Wei Pan, Daniel R. Storm, Zhengui Xia

**Affiliations:** 1 Graduate Program in Molecular and Cellular Biology, University of Washington, Seattle, Washington, United States of America; 2 Department of Pharmacology, University of Washington, Seattle, Washington, United States of America; 3 Toxicology Program in the Department of Environmental and Occupational Health Sciences, University of Washington, Seattle, Washington, United States of America; Institut National de la Recherche Agronomique-CNRS UMR6175, France

## Abstract

Adult neurogenesis in the dentate gyrus of the hippocampal formation has been implicated in several forms of hippocampus-dependent memory. However, its role in the persistence of remote memory is unknown. Furthermore, whether the hippocampus plays a role in maintaining remote contextual memories is controversial. Here we used an inducible gene-specific approach for conditional deletion of *erk5* in the adult neurogenic regions of the mouse brain to specifically impair adult neurogenesis. The *erk5* gene was conditionally deleted under three different experimental conditions: prior to training for contextual fear, 6 days after training, or 5 weeks after training, We present evidence that remote memory was impaired under all three conditions. These data demonstrate that ongoing adult neurogenesis is required both for the initial establishment and the continued maintenance of remote contextual fear memory, even after the remote memory has transferred into extra-hippocampal regions of the brain 5 weeks after training.

## Introduction

Adult neurogenesis occurs in distinct regions in the mammalian brain under normal physiological conditions including the subgranular zone (SGZ) in the dentate gyrus of the hippocampal formation [1]. Adult neurogenesis in the SGZ has been implicated in several forms of hippocampus-dependent learning and memory [2]. We previously reported that ERK5 expression in the adult brain is limited to the neurogenic regions and that adult neurogenesis in the SGZ is regulated by the ERK5 MAP kinase both *in vitro* and *in vivo* [3]. Interestingly, deletion of *erk5* specifically in the adult neurogenic regions impairs adult neurogenesis in the hippocampus [3], and disrupts the more demanding forms of hippocampus-dependent memory including contextual fear memory generated by a weak foot shock as well as contextual fear extinction [4].

Whether the maintenance of remote contextual memory depends on the hippocampal formation is controversial with some evidence supporting its involvement [5]–[7]. However, other data have suggested that the hippocampus only plays a temporary role in the formation of new contextual fear memories and that remote memories are independent of the hippocampus and are permanently stored in extra-hippocampal regions such as the neocortex [8]–[12]. Furthermore, although adult neurogenesis occurs throughout adult life in the dentate gyrus and may modulate the duration of hippocampus-dependent memory [13], its role in the maintenance of remote memory has not been reported. Here, we present evidence that remote contextual memory depends upon ongoing adult neurogenesis even after remote memory is established.

## Materials and Methods

### Ethics Statement

All animals used in this study were approved by the University of Washington Institutional Animal Care and Use Committee. Experimental conditions and procedures were performed with direct approval under protocol 3041-04.

### Animals

The generation of Nestin-CreER™/ERK5^loxP/loxP^ mice has been previously described [4]. All experiments were performed using identically handled littermates. Standard housing conditions were applied (12 h light/dark cycle) with food and water provided *ad libitum.* All experimental procedures were approved and performed in accordance with the guidelines under the University of Washington Institutional Animal Care and Use Committee.

### Administration of Tamoxifen

**Figure 1:**
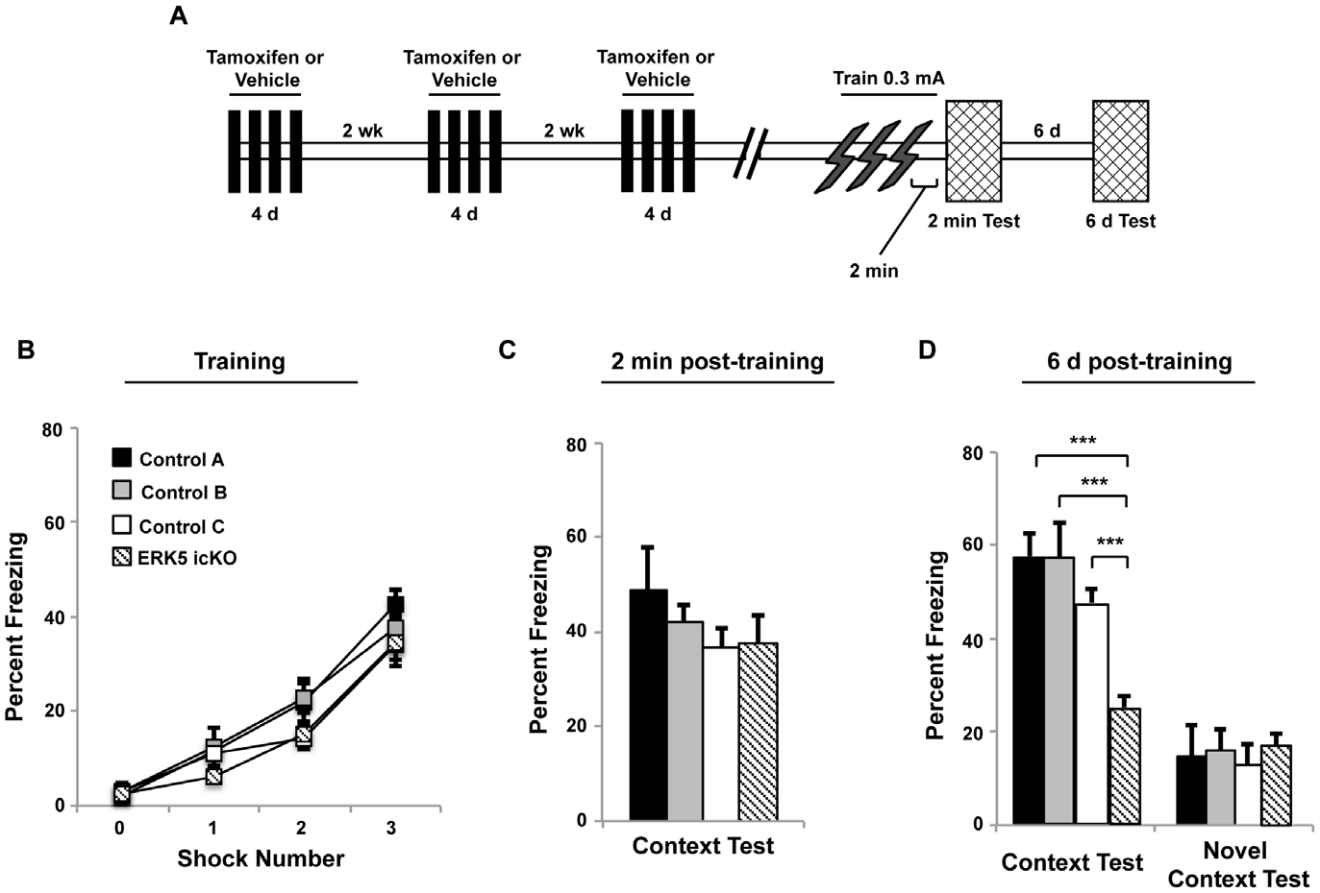
ERK5 icKO mice display normal shock sensitivity, acquisition and retrieval of contextual fear memory, yet show reduced contextual fear memory 6 d after training with the 3 × 0.3 mA foot shock paradigm. A) Schematic depiction of the experimental design. B) Freezing behavior displayed by ERK5 icKO and control mice immediately following each of the 3 successive 0.3 mA foot shocks during training. C) ERK5 icKO mice have acquired and can retrieve contextual fear memory 2 min after training. D) ERK5 icKO mice show reduced contextual fear memory 6 d after training while freezing behavior in the novel context was unaffected.

**Figure 2:**
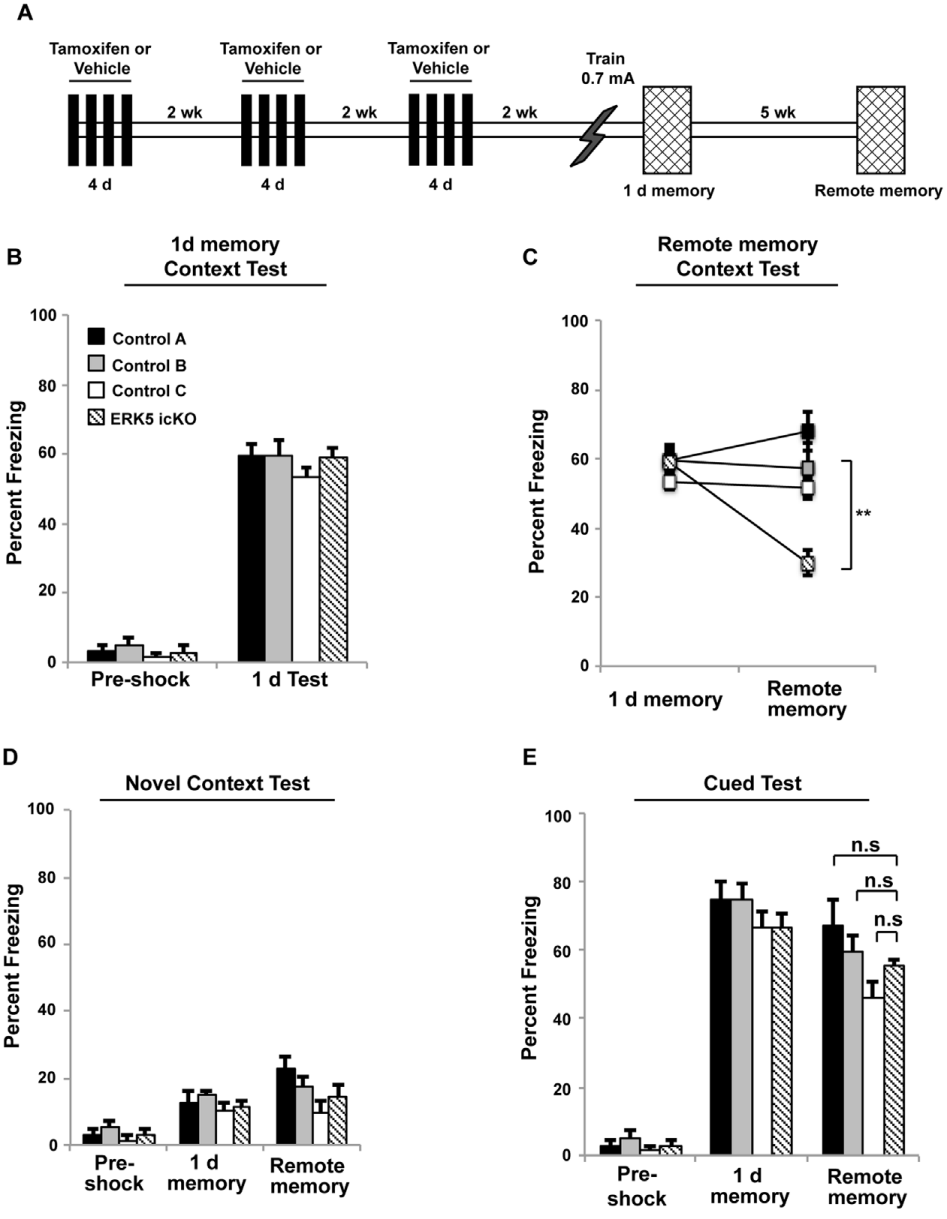
ERK5 icKO mice are impaired in remote memory when the *erk5* gene was inducibly deleted prior to training. A) Schematic depiction of the experimental design. Mice were treated with tamoxifen to delete *erk5* before contextual fear conditioning with the 1×0.7 mA foot shock paradigm. B) Freezing behavior in the shocking context 1 d after training does not differ between ERK5 icKO and control mice. C) Remote contextual memory is impaired in ERK5 icKO mice when tested 5 wks post-training. D) Minimal freezing behavior is observed in a novel context at both 1 d and 5 wks post-training for all treatment groups. E) Amygdala-dependent, cued-fear conditioning is normal in ERK5 icKO mice at both 1 d and 5 wks post training.

**Figure 3:**
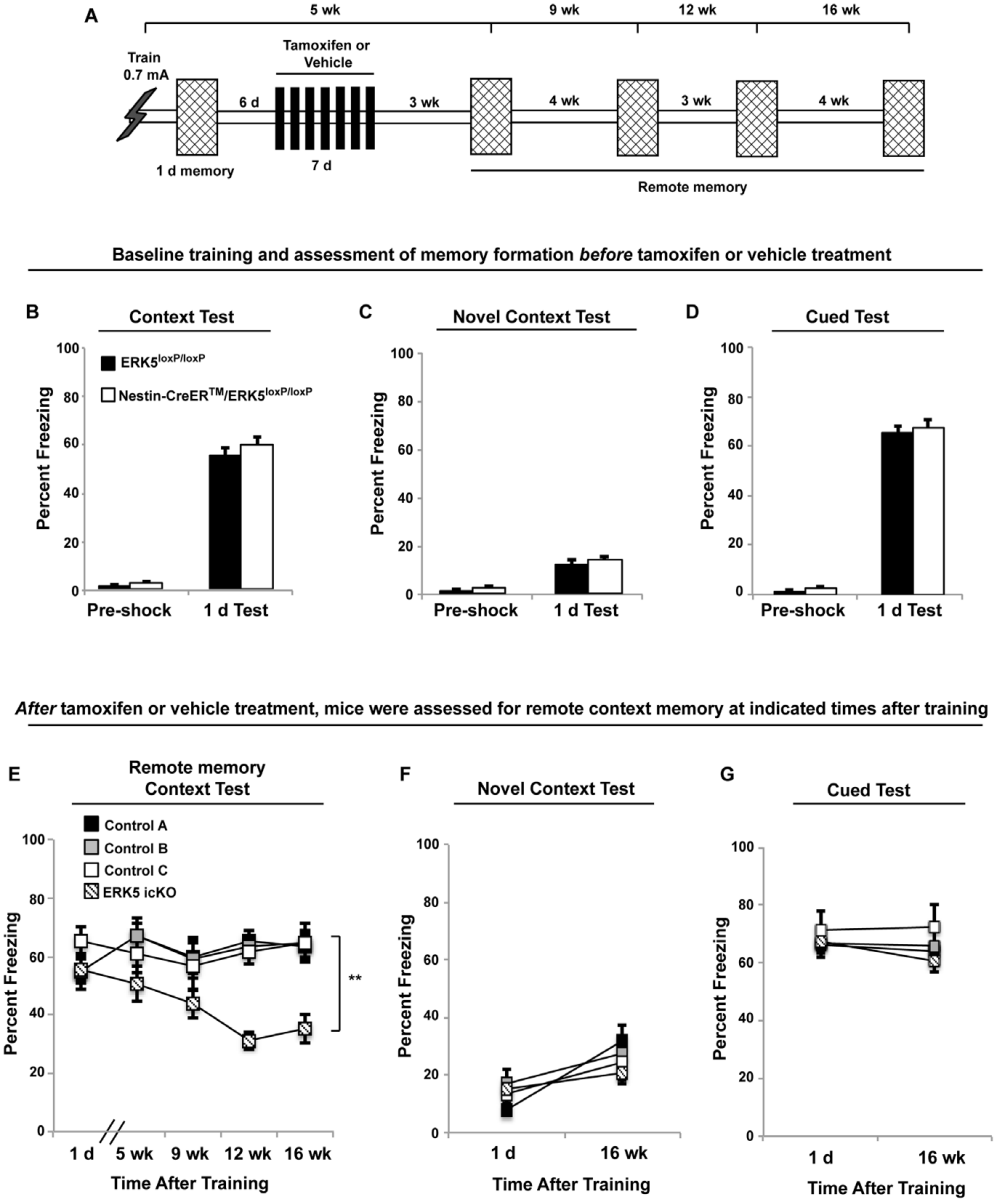
Deletion of the *erk5* gene 6 days after training causes decay of remote contextual fear memory. A) Schematic depiction of the experimental design. B–D) Assessment of memory formation for contextual fear conditioning (B), novel context (C), and cued fear (D) before *erk5* deletion. Mice were trained with the 1×0.7 mA foot shock paradigm and freezing behavior was assessed 1 d after training. E–G) Six days after training, mice were treated with tamoxifen for 7 d to delete *erk5* or with vehicle control. Freezing behavior was assessed in the training context (E), a novel context (F), or in a novel context paired with the cue (G) at 5, 9, 12, and 16 wks after training.

**Figure 4:**
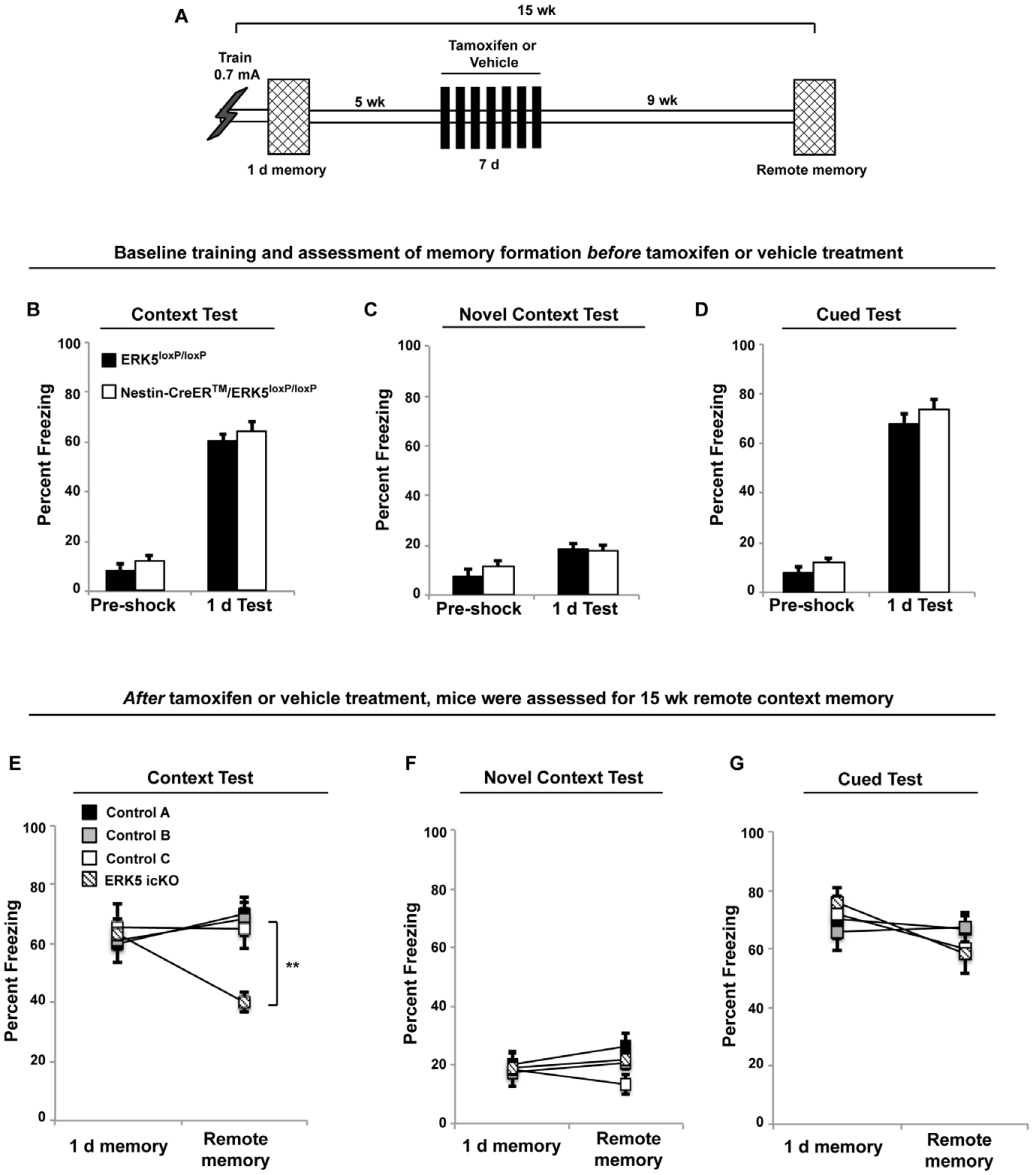
Deletion of the *erk5* gene 5 weeks post-training impairs remote memory. A) Schematic depiction of the experimental design. B–D) Assessment of memory formation for contextual fear conditioning (B), novel context (C), and cued fear (D) before *erk5* deletion as in Figure 3. E–G) Five weeks after training, mice were treated with tamoxifen for 7 d to delete *erk5* or with vehicle control. Remote memory for the shocking context was significantly reduced 15 wks after training in ERK5 icKO mice (E), while their response to a novel context (F) or the cue (G) was unaffected.

Male and Female 10–12 week old adult mice were used. Tamoxifen was freshly dissolved in 2% glacial acetic acid and corn oil (Sigma) at 37°C and vortexed periodically. To initiate Cre-mediated *erk5* gene deletion, 5 mg of pre-warmed tamoxifen was delivered via oral gavage by either of two paradigms: A) once per day for 4 d in each cycle for 3 total cycles (3 × 4 d) with a 2 week inter-cycle interval for Figures 1 and 2, or B) once per day for 7 d for Figures 3 and 4. Vehicle control animals were dosed in an identical manner with corn oil solution.

### Fear Memory Conditioning Using a Weak Foot Shock

This was performed as described [4]. Briefly, The foot shock context is a 10″(W)×10′′(D)×16′′(H) square-shaped box fitted with metal grid shock floor (Coulbourn). On training day, mice were placed in this context box with striped wall paper and allowed to habituate to the environment for 2 min. Immediately following habituation, animals were subjected to 3 successive foot shocks, 0.3 mA, 2 s each and separated by 2 min inter-shock intervals. Freezing behavior was scored immediately after each shock for 2 min. Mice were returned to their home cages for a 2 min period while the context box was cleaned, and then back to the cleaned context box for 2 min, during which time their freezing behavior was assessed as a measure of contextual fear memory acquisition. Six days post-training, mice were brought back to the context box for 2 min and freezing behavior scored. Two hours later, mice were placed in a novel context (hexagonal shaped arena with clear side walls) in a different room and assessed for freezing behavior for 2 min. Freezing behavior is defined by lack of bodily movement with all 4 paws on the grid floor except for normal respiration.

### Remote Memory Assessment

Mice were trained for contextual fear and cued-fear conditioning using the standard 1×0.7 mA foot shock paradigm as described [4]. The establishment of contextual fear memory was confirmed by scoring freezing behavior in the context box for 2 min at 24 h after training. Freezing behavior in response to the cue or in a novel environment was also scored. Remote memory was assessed under three different conditions: A) *erk5* was deleted prior to training, B) *erk5* was deleted 6 d after training, and C) *erk5* was deleted 5 wks following training. In all cases, remote contextual and cued-fear memories, as well as freezing behavior in a novel context were analyzed as described [4].

### Statistical Analysis

Two-way ANOVA with repeated measures was used to analyze data in Figure 3E. One-way ANOVA with Fisher’s LSD *post-hoc* analysis was performed for all remaining data. Data represent mean ± standard error of means (s.e.m.). *, p<0.05; **, p<0.01; ***, p<0.001; n.s., not statistically significant (p≥0.05). n ≥6 for each genotype/control in each experiment.

## Results

### Conditional Deletion of *erk5* Causes Deficits in Contextual Memory Consolidation but does not Affect Memory Acquisition or Retrieval

Contextual fear memory is significantly reduced in the inducible and conditional ERK5 knockout (icKO) mice when they are trained using a challenging weak shock protocol (3×0.3 mA foot shocks, 2 s each) but not when trained with a stronger foot shock (1×0.7 mA. 2 s) [4]. The deficit in contextual memory when mice are trained with 3×0.3 mA foot shocks could result from impairments in memory acquisition, retrieval, or consolidation. To address this issue, we employed the ERK5 icKO mice in which tamoxifen treatment (3×4 d) of adult Nestin-CreER™/ERK5^loxP/loxP^ mice selectively deletes the *erk5* gene in Nestin-expressing adult neural stem cells [3]. This leads to significant impairment of adult neurogenesis quantified by the reduction of the total number of BrdU, NeuN double-positive adult-born neurons in the SGZ [3]. Three groups of control mice were used: ERK5^loxP/loxP^ mice treated with either vehicle control (control A) or tamoxifen (control B), and Nestin-CreER™/ERK5^loxP/loxP^ mice treated with vehicle control (control C) (Table 1). When ERK5 icKO and control mice were trained for contextual fear conditioning using the weak shock paradigm (Fig. 1A), they all displayed similar degrees of freezing immediately after each foot shock during training, indicating comparable levels of shock sensitivity (Fig. 1B). Mice were then tested for contextual fear memory at 2 min and 6 d after training. ERK5 icKO mice behaved similarly as control mice when tested 2 min after training (Fig. 1C), indicating that they can acquire and retrieve contextual fear memory. However, ERK5 icKO mice exhibited significantly less freezing at 6 d post-training (Fig. 1D). The contextual fear memory at 6 d was context-specific since all mice froze minimally when exposed to a novel context. Consolidation of contextual fear memory in mice occurs within 24 h and continues thereafter. Our data suggest that the impaired contextual fear memory of ERK5 icKO mice 6 d after training using the weak foot shock paradigm is not due to a deficit in acquisition or memory retrieval, but rather consolidation and persistence of contextual fear memory.

**Table 1:** Animal genotypes and treatment.

Name	Genotype	Treatment
Control A	ERK5^loxP/loxP^	Vehicle
Control B	ERK5^loxP/loxP^	Tamoxifen
Control C	Nestin-CreER™/ERK5^loxP/loxP^	Vehicle
ERK5 icKO	Nestin-CreER™/ERK5^loxP/loxP^	Tamoxifen

### Targeted Deletion of *erk5* Prior to Training for Contextual Fear Conditioning Interferes with the Establishment of Remote Memory

Since ERK5 icKO mice exhibited impaired contextual fear memory 6 d after training using the 0.3 mA foot shock training paradigm (Fig. 1), we employed a commonly used, stronger shock training protocol (0.7 mA, 2 s) to assess the importance of adult neurogenesis for remote memory (Fig. 2A). ERK5 icKO and control mice exhibited equivalent levels of contextual fear memory 1 d after training (Fig. 2B). When assessed for remote contextual fear memory 5 wks post-training, however, ERK5 icKO mice exhibited significantly reduced memory (Fig. 2C). This result was context-specific since all mice froze minimally when exposed to a novel context (Fig. 2D). Additionally, all mice froze at similar levels when assessed for cued-fear conditioning at 5 wks, suggesting that amygdala-dependent fear memory was intact in ERK5 icKO mice (Fig. 2E). These data suggest that adult neurogenesis is required for the establishment of remote contextual fear memory.

### Conditional Deletion of *erk5* Six Days After Training for Contextual Fear Conditioning Causes Remote Memory Decay

We next examined if new neurons born after initial acquisition and consolidation of contextual fear memory are necessary for the expression of remote memory. Mice were first trained and assessed 1 d after training for context-specific and cued-fear conditioning (Fig. 3A–D). Both ERK5^loxP/loxP^ and Nestin-CreER™/ERK5^loxP/loxP^ mice performed similarly suggesting that prior to tamoxifen treatment, no phenotypic differences exist. After mice had 6 d to consolidate contextual fear memory, they were treated with vehicle control or tamoxifen for 7 d to delete *erk5* thereby inhibiting adult neurogenesis. This treatment has been demonstrated previously to significantly reduce the total number of cells expressing ERK5, NeuroD, as well as BrdU and NeuN double-positive adult-born neurons in the SGZ [4]. Remote contextual memory was assessed at 5, 9, 12, and 16 wks after initial training. ERK5 icKO mice froze significantly less than control mice, especially at 12 and 16 wks post-training (Fig. 3E). This effect was context-specific and did not affect cued-fear conditioning (Fig. 3F, G).

### Ongoing ERK5-regulated Adult Neurogenesis is Required for the Maintenance of Remote Memory, Even After Remote Memory has been Established

Another important question is whether ongoing adult neurogenesis is necessary for the maintenance and expression of remote contextual fear memory after the memory has transferred into extra-hippocampal regions, which usually takes approximately 4 wks in mice [5], [13], [14]. To address this question, ERK5^loxP/loxP^ and Nestin-CreER™/ERK5^loxP/loxP^ mice were trained as in Figure 3 with 0.7 mA foot shock and their contextual fear memory tested and confirmed 1 d after training (Fig. 4A–D). Both groups of mice performed equally well after training. Five weeks after training, we treated mice with vehicle control or tamoxifen to delete *erk5* and disrupt adult neurogenesis. Mice were tested for context-specific freezing behavior 15 wks after training. Interestingly, ERK5 icKO mice froze significantly less than control mice when assessed for persistence of remote contextual memory 15 wks post-training (Fig. 4E), but not in remote cued-fear memory or in the novel context (Fig. 4F, G). These data suggest a context-specific deficit in the maintenance of remote contextual fear memory even when *erk5* was deleted in adult neurogenic regions after remote memory had been established.

## Discussion

Deficits in learning and memory are prominent features of many mental disorders. Understanding molecular mechanisms underlying learning and memory are key to the development of therapies to correct cognitive disorders in the treatment of mental illness. The goal of this study was to investigate the role for ERK5 MAP kinase and ERK5-regulated adult neurogenesis in the memory formation of contextual fear and the maintenance of this memory.

We previously reported that contextual fear memory is significantly reduced in ERK5 icKO mice at 24 h after animals were trained using a challenging training protocol (3×0.3 mA foot shocks) [4]. Here, we demonstrate that under the same conditions, ERK5 icKO mice showed similar contextual fear memory as control mice when tested 2 min after training. Thus, the reduced contextual fear memory seen at 24 h using the 3×0.3 mA foot shocks training protocol is most likely due to a deficit in consolidation of the fear memory rather than deficits in memory acquisition and/or retrieval.

Since inhibition of adult neurogenesis may increase anxiety [15], it is possible that the deficits in remote contextual fear memory observed in ERK5 icKO mice may be due to increased anxiety. However, ERK5 icKO mice are not deficient in short-term (2 min, 1 day) contextual fear memory, nor in both short-term (1 day) and remote (5 wks, 15 wks, and 16 wks) cued fear memory. If increased anxiety significantly affects remote contextual fear memory, then it should have also affected short-term contextual and cued fear memory, as well as remote cued fear memory. Therefore, it seems unlikely that the observed defect of remote memory in ERK5 icKO mice is due to increased anxiety.

It is generally agreed that contextual fear memory is initially stored in the hippocampus for the short term. Furthermore, interaction between the hippocampus and neocortex after the formation of recent memory is crucial for the storage of remote memory regardless of the exact storage location for remote memory [9], [16], [17]. Indeed, post-training deletion of *erk5*, which reduces adult neurogenesis [3], during the hippocampus-dependent period (6 d post-training) caused retrograde amnesia of contextual fear memory. Together with a recent report using diphtheria toxin-based strategy to ablate mature, adult-generated neurons within the week after training [18], these data suggest that interference with adult neurogenesis can cause retrograde amnesia.

Whether the hippocampal formation is required for the maintenance of remote contextual memory once the memory is established is controversial [5]–[12], [14], [19]–[21]. Furthermore, before this study, it was not known if continuing adult neurogenesis plays a role in the expression and maintenance of remote contextual memory. For example, one study suggests that adult neurogenesis modulates the duration of hippocampus-dependent period of associative fear memory but inhibition of adult neurogenesis does not cause loss or reduction of remote memory *per se* [13]. Using our transgenic mouse line, we were able to specifically and conditionally target *erk5* in neural stem/progenitor cells in adult neurogenic regions, thus interfering with adult neurogenesis [3], [4] to assess the importance of adult-born neurons in the hippocampus for the expression and persistence of remote memory. Our data indicate a critical role for adult-born neurons in both the expression and maintenance of remote contextual memory using three different paradigms, exemplified with observed deficits when *erk5* was conditionally deleted prior to training, 6 d after training, or 5 wks after training.

The cortex and basolateral amygdala are required for remote contextual fear memory [9], [22], [23]. It is possible that the functional connectivity between the hippocampal formation and amygdala or cortex during memory retrieval and reconsolidation is important for the maintenance of remote contextual fear memory. Although this study does not directly address this functional connectivity, it is important to note that ERK5 is selectively expressed in the neurogenic regions but not other regions of the adult brain [3], [4]. Furthermore, the *erk5* gene deletion is restricted to neurogenic regions of the adult brain [3], [4]. Data presented in this study clearly suggest that the continual formation of new neurons in the dentate gyrus is integral for the establishment and maintenance of remote contextual fear memory, even after the memory has transferred out of the hippocampus. Our data support the general hypothesis that the hippocampus has a long-term role in the continued expression of contextual fear memory [24], [25], and may have important implications for the treatment of memory disorders.
